# Supplementary material for: Fungal β-glucan-facilitated cross-feeding activities between Bacteroides and Bifidobacterium species
Source: Commun Biol. 2023 May 30;6:576. doi: 10.1038/s42003-023-04970-4 (PMC10229575; doi:10.1038/s42003-023-04970-4)
Supplement: Supplementary file 2 — Supplementary Material [file 42003_2023_4970_MOESM2_ESM.pdf]

SUPPLEMENTARY INFORMATION

Fungal β-glucan-facilitated cross-feeding activities between Bacteroides and Bifidobacterium species.

Pedro Fernandez-Julia[1], Gary W. Black[1], William Cheung[1], Douwe Van Sinderen[2], Jose Munoz-Munoz[1*]

Table S1. *Bacteroides* species tested with all substrates.

	Mycoprotein β-glucan	Yeast β-glucan
Ba. thetaiotaomicron VPI-5482		
Ba. cellulosilyticus WH2		
Ba. cellulosilyticus DSM 14838		
Ba. ovatus ATCC 8483		
Ba. intestinalis DSM 17393		
Ba. fragilis ATCC 25285		
Ba. finegoldii DSM 17565		
Ba. vulgatus ATCC 8482		
Ba. caccae ATCC 43185		
Ba. clarus DSM22519		
Ba. dorei DSM17855		
Ba. egerthii DSM 20697		
Ba. fluxus DSM 22534		
Ba. massiliensis DSM 17679		
Ba. oleiciplenus DSM 22535		
Ba. plebeius DSM 17135		
Ba. salyersae DSM 18765		
Ba. stercolis ATCC 43183		
Ba. uniformis ATCC 8492		
Ba. xylanosolvens XB1A		
Ba. intestinihominis DSM 21032		

Dysgonomonas gadei ATCC BAA-286		
Dysgonomonas mosii DSM 22836		

Green= full growth (OD$_{600}$ 0.9-1.5), Yellow= medium growth (OD$_{600}$=0.4-0.8) and Red= no or low growth (OD$_{600}$=0-0.3). The time for all growths was 24h and all experiments were obtained in triplicates.

Table S2. Primers used in this study.

	Sequence
Baccell WH2	
Bccell WH2_01926 (GH3)	Forward: GCGGCGGCTAGCCAGGAAAAGGCAAATACC<br>Reverse: GCGGCGCTCGAGCTATTGTAAGACAGTAAA
Bccell WH2_01931 (GH157)	Forward: GCGGCGGCTAGCCAATTCAGTTCTTCTCCG<br>Reverse: GCGGCGCTCGAGTCATGTCAGCAACATCTT
Bccell WH2_02537 (GH30_3)	Forward: GCGGCGGCTAGCTGCGGATCGGACCACAAT<br>Reverse: GCGGCGCTCGAGTTATTTCCACTTGAAAGA
Bccell WH2_02541 (GH2)	Forward: GCGGCGGAATTCCAACATGAGAGCAAAACG<br>Reverse: GCGGCGCTCGAGTTAATAAAGTTTCCTGAG
Primers for qPCR cross feeding	Forward: 5′ -AGCAGGCGGAATTCGATAAG-3′<br>Reverse: 5′ -GTGTACAGTGCCAGGCATAA-3′
BT	
BT3312 (GH30_3)	Forward: GCGGCGGCTAGCAATAGTGATGATGCGGAA<br>Reverse: GCGGCGCTCGAGTTACTTTGACTTTGCCCAACG
BT3314 (GH3)	Forward: GCGGCGGCTAGCCAGACACCAGTATATCTA<br>Reverse: GCGGCGCTCGAGCTATCTCAACTCAAATGG
Primers for qPCR cross feeding	Forward: 5′ -AGGTGCAGGCAACCT-3′<br>Reverse: 5′ -AATTCCCGTTCTCCATGTCC-3′
Bi. breve UCC2003	
Bbr_0109 (GH1)	Forward: GCGGCGGCTAGC ATGACATTCGTTTTTCCG<br>Reverse: GCGGCGCTCGAG TCA GAT TCC TTC CTG GAT
Primers for qPCR cross feeding	Forward: AGAGTTTGATCCTGGCTCAG<br>Reverse: CTACGGCTACCTTGTTACGA
Bi. longum subsp. *longum*	

Primers for qPCR cross feeding	Forward: AGAGTTTGATCCTGGCTCAG<br>Reverse: CTACGGCTACCTTGTTACGA
Bi. longum subsp. *infantis*	
Primers for qPCR cross feeding	Forward: AGAGTTTGATCCTGGCTCAG<br>Reverse: CTACGGCTACCTTGTTACGA
Lb. plantarum	
Primers for qPCR cross feeding	Forward: CAC CGC TAC ACA TGG AG<br>Reverse: CCA CCG CTA CAC ATG GAG TTC CAC T

Figures

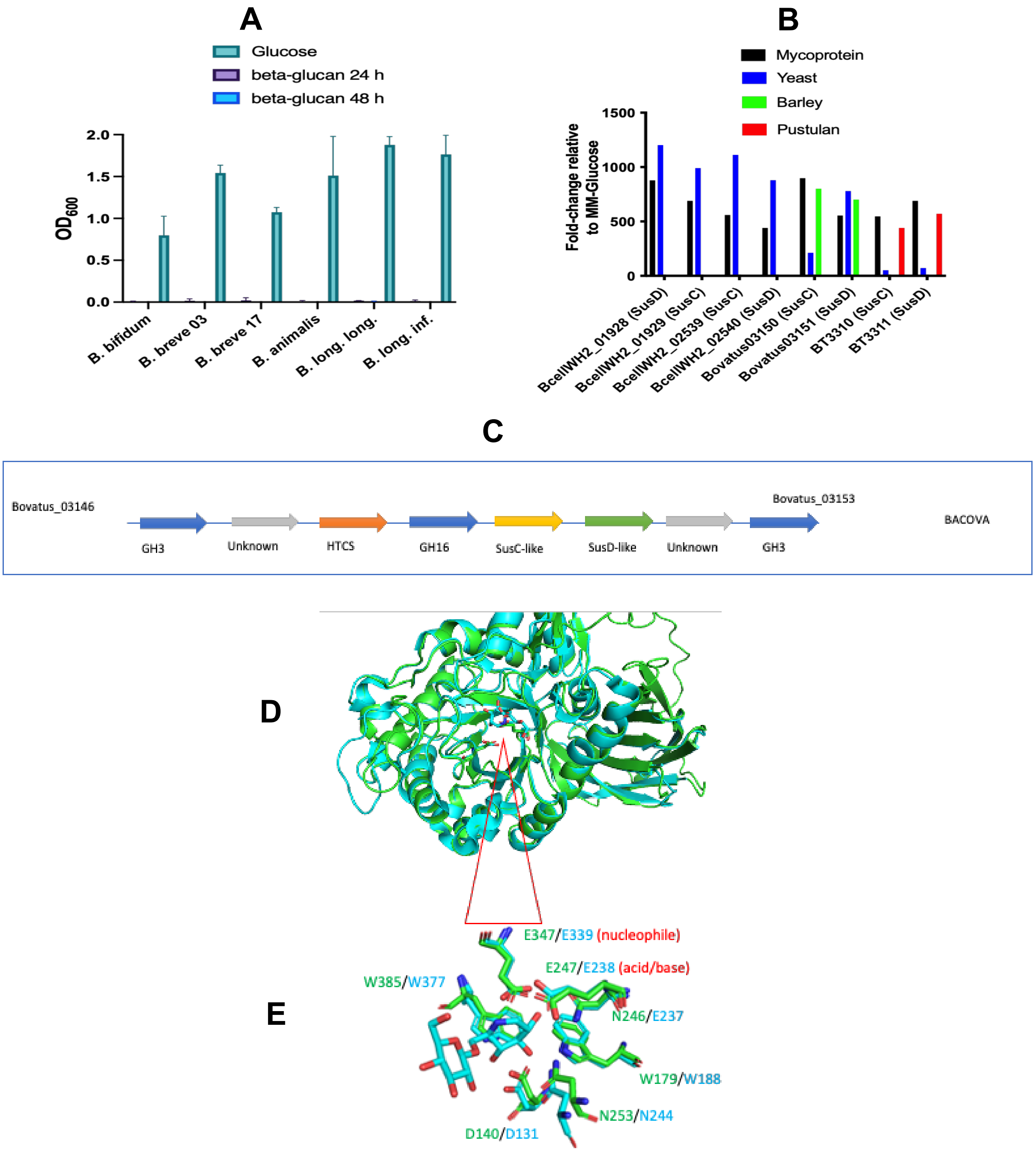

Fig. S1. Growth of *Bifidobacterium* sp. on intact polysaccharides and structural analysis of the different enzymes involved in different GULs. All growth and qPCR experiments have been produced in 3 different independent replicates (n=3). **A.** Ability of *Bifidobacterium* to use glucose, mycoprotein and yeast β-glucan as carbon source. **B.** qPCR analysis of SusC/D pairs in Baccell WH2, Bacova and BT when grown on mycoprotein, yeast, barley and pustulan β-

glucan as carbon source comparing with glucose as monosaccharide. We selected the relevant pairs according to proteomic and bioinformatics analysis, showing the upregulation of the SusC/D pairs in the GULs. **C.** GUL structure in Bacova acting on barley β-glucan. **D.** Structural alignment of GH30_3 active on β-glucan. BT3312 (Cyan) and BcellWH2_02537 (green). **E.** Conservation of key residues in the active site; BT3312 (Cyan) and BcellWH2_02537 (green).

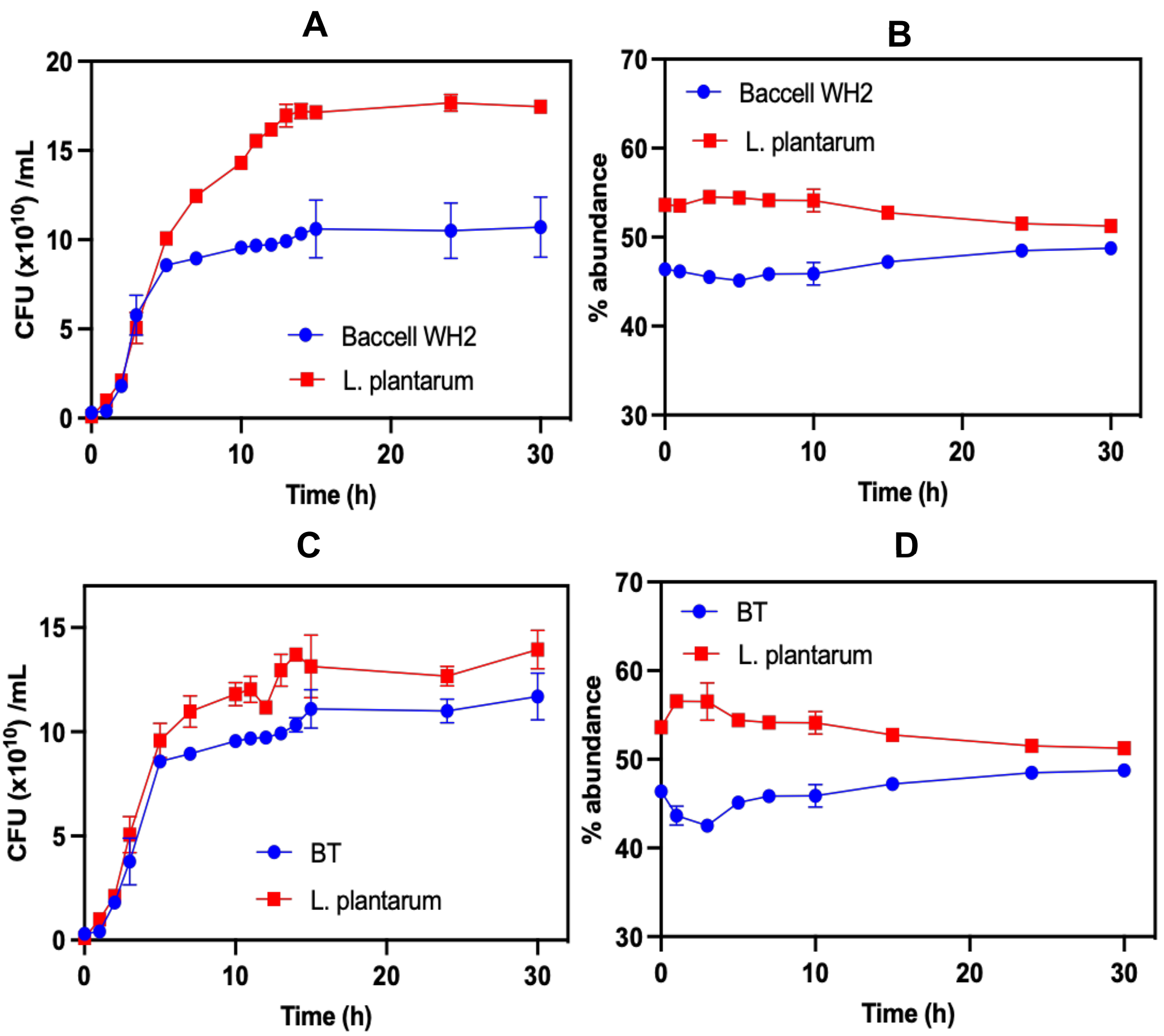

Fig. S2. Cross-feeding experiments between Baccell WH2 and BT and *Lactiplantibacillus spp*. **A.** Colony forming units of Baccell WH2 + *Lactiplantibacillus plantarum*. **B.** Percentage of Baccell WH2 + *Lactiplantibacillus plantarum.* **C.** Colony forming units of BT + *Lactiplantibacillus plantarum*. **D.** Percentage of BT + *Lactiplantibacillus plantarum.* All cross-feeding experiments have been produced in 2 different independent replicates (n=2).

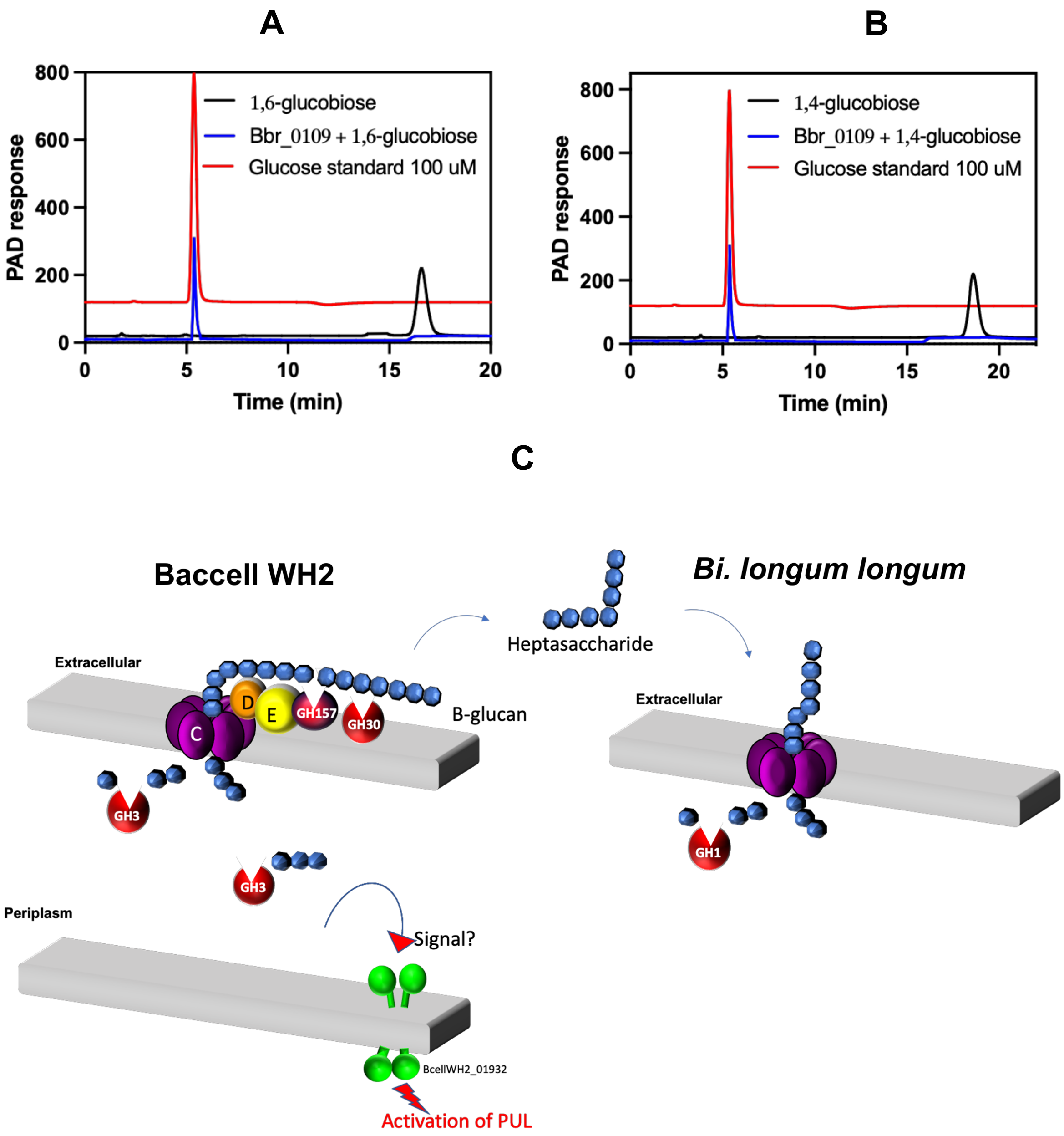

Fig. S3. Biochemical analysis of Bbr_0109 with different disaccharides and general model for the cross-feeding interactions. **A-B.** HPLC of the Bbr_0109 activity with 1,6-glucobiose (A) and 1,4-glucobiose (B), respectively. All HPLC experiments have been produced in 3 different independent replicates (n=3). **C.** General model for the degradation of fungal β-glucan by Baccell WH2 and cross-feeding with *Bifidobacterium.*

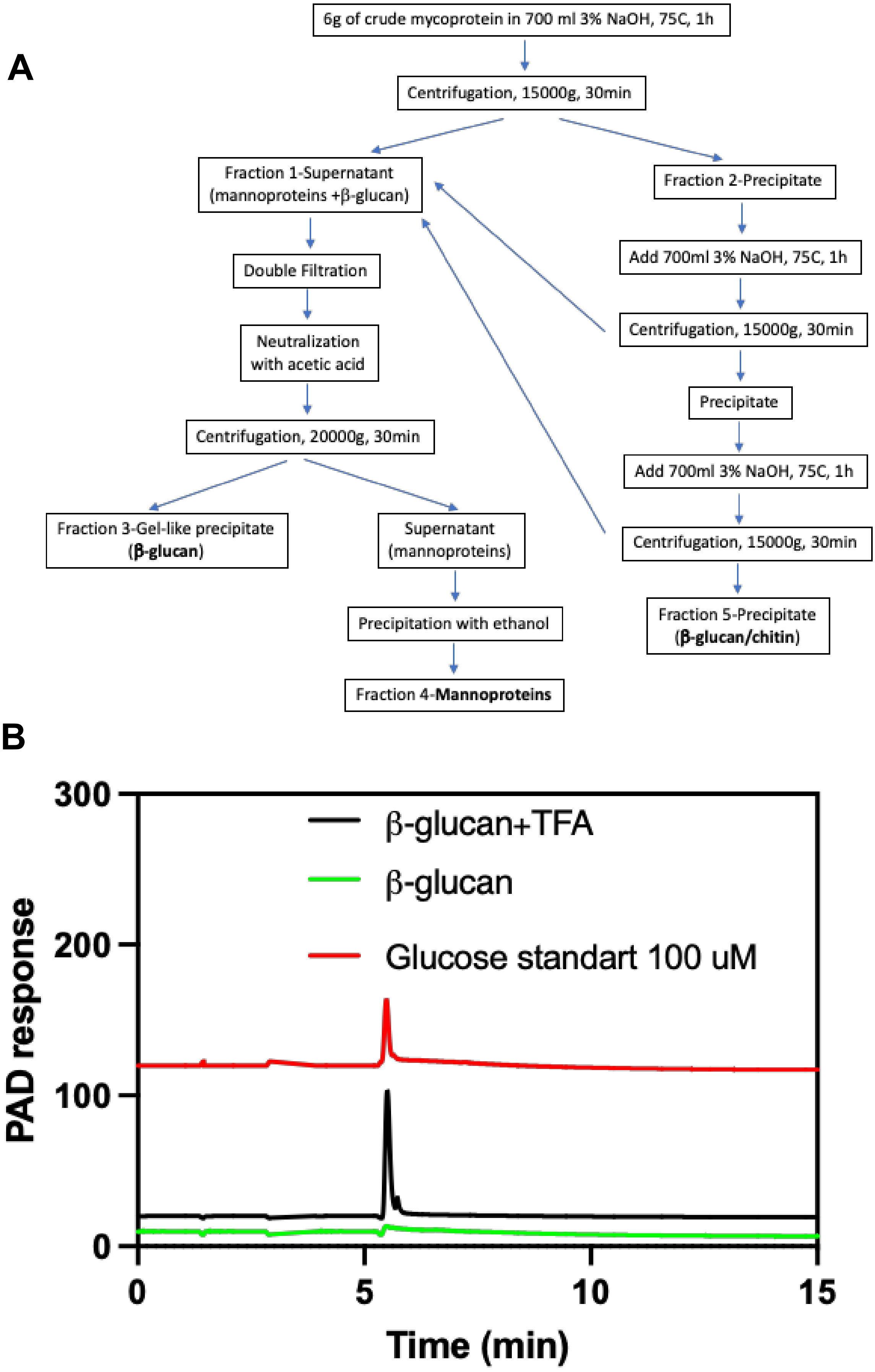
A
6g of crude mycoprotein in 700 ml 3% NaOH, 75C, 1h
Centrifugation, 15000g, 30min
Fraction 1-Supernatant (mannoproteins +β-glucan)
Fraction 2-Precipitate
Double Filtration
Add 700ml 3% NaOH, 75C, 1h
Centrifugation, 15000g, 30min
Neutralization with acetic acid
Precipitate
Centrifugation, 20000g, 30min
Add 700ml 3% NaOH, 75C, 1h
Fraction 3-Gel-like precipitate (β-glucan)
Supernatant (mannoproteins)
Centrifugation, 15000g, 30min
Precipitation with ethanol
Fraction 5-Precipitate (β-glucan/chitin)
Fraction 4-Mannoproteins
B
β-glucan+TFA
β-glucan
Glucose standart 100 uM
PAD response
300
200
100
0
0
5
10
15
Time (min)

Fig. S4. Analysis of the β-glucan extraction from mycoprotein. **A.** Purification protocol for fungal β-glucan extraction. **B.** HPLC of extracted fungal β-glucan with acid hydrolysis (HCl 2M) to confirm the purity of the sample.

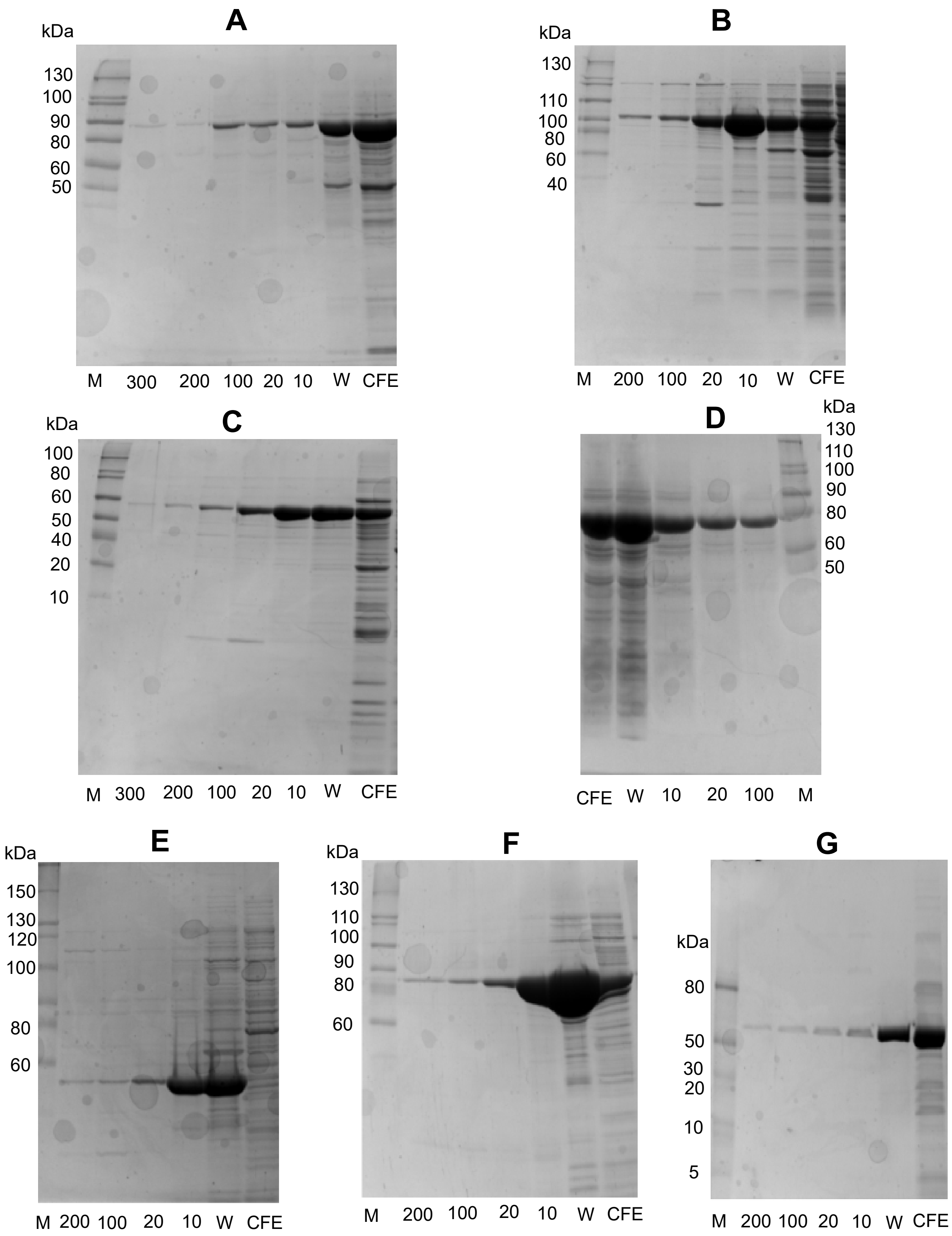

CFE; Cell Free Extract W; Waste 10; 10 mM Imidazole 20; 20 mM Imidazole
100; 100 mM Imidazole 200; 200 mM Imidazole 300; 300 mM Imidazole M; Marker

Fig. S5. SDS-gels of the recombinant proteins expressed in this study and the theoretical molecular weight for each of them. **A.** BcellWH2_01926 (GH3, Mw 85423); **B.**

BcellWH2_02541 (GH2, Mw 102478); **C.** BcellWH2_02537 (GH30_3, Mw 57669); **D.** BcellWH2_01931 (GH157, Mw 73658); **E.** BT3312 (GH30_3, Mw 55468); **F.** BT3314 (GH3, Mw 84558) and **G.** Bbr_0109 (GH1, Mw 52130).
